# The Impact of the COVID-19 Pandemic on Smoking Consumption: A Systematic Review of Longitudinal Studies

**DOI:** 10.3389/fpsyt.2022.941575

**Published:** 2022-07-12

**Authors:** Nerea Almeda, Irene Gómez-Gómez

**Affiliations:** Department of Psychology, Universidad Loyola Andalucía, Sevilla, Spain

**Keywords:** COVID-19, smoking, prospective cohort studies, systematic review, mental health, tobacco

## Abstract

The COVID-19 pandemic has generated a global health crisis that has negatively impacted the mental health and wellbeing of the population. A large amount of scientific literature has emerged since 2019, but none of these studies have focused on assessing the impact of COVID-19 on smoking consumption. We aimed to analyse the changes in smoking consumption during the COVID-19 pandemic through longitudinal studies. This systematic review follows the PRISMA Statement. This study was registered on PROSPERO (CRD42021282235). MEDLINE, ERIC, PsycARTICLES, Scopus, Web of Science and PsycINFO databases were searched from inception to 24 October 2021. We completed an extensive assessment of all prospective cohort studies that aimed to explore the effect of the COVID-19 pandemic on tobacco consumption habits. According to the PICOS's acronym, we included all population (P) types and studies developed before and during the COVID-19 pandemic (I) with a change in nicotine consumption as the outcome (O), as well as prospective cohort studies. The risk of bias was assessed using the Newcastle–Ottawa Scale for observational studies. The results showed that 14 cohorts reported in 11 articles fulfilled the inclusion criteria. A total of 58,052 participants were included in the review. Most of the studies pointed out a reduction in the number of cigarettes and e-cigarettes consumed from baseline (before the pandemic) to follow-up (during the pandemic). Only two studies reported an increase in cigarette or e-cigarette consumption from baseline to follow-up. The majority of studies presented a low risk of bias. In conclusion, the impact of the COVID-19 pandemic on smoking behavior is complex and uncertain. The decrease in smoking consumption during the pandemic could be related to the fear of becoming infected by COVID-19, the advancement of COVID-19, and the reduction in social gatherings. In several cases, the increases in nicotine consumption can be explained by psychological distress. These findings can be used to create strategies to prevent relapses during the post-vaccination phases of the pandemic.

**Systematic Review Registration:** PROSPERO, identifier: CRD42021282235.

## Introduction

The novel coronavirus disease (COVID-19) was identified for the first time in Wuhan (China) on 31 December 2019 (1). For over 2 years, the global pandemic caused 528,816,317 confirmed cases of COVID-19 and 6,294,969 deaths on 1 June 2022, while 11,947,644,522 vaccine doses have been administered worldwide (2). During these 2 years, the governments declared lockdown and imposed several containment measures (quarantine, isolation and social distancing) to detain the spread of the virus (3–5). These measures have detained the virus contagion and have generated a worsening in mental health and impacted health behaviors (6–8).

Due to containment measures, lockdowns and the global crisis caused by the pandemic, the population has inevitably suffered from changes in health behaviors such as sleep, substance abuse, physical activity and diet, which could have long-term consequences on global health (9). It is worth highlighting that the COVID-19 pandemic has caused more than a health crisis, which has impacted society and the economy, increasing poverty and inequalities globally (10). The pandemic has also generated psychosocial and socioeconomic insecurities that impacted wellbeing (11). In this sense, socioeconomic conditions such as deprivation and low-income increase perceived stress levels and, consequently, the development of risk behaviors such as daily smoking (12).

Considering that the COVID-19 pandemic has pushed people worldwide to change their daily lives and sociodemographic conditions (loss of job and income) and has raised their mental health conditions, it is expected that the coronavirus has also had an impact on health risk behaviors such as smoking consumption. Scientific literature points out that the relationship between smoking consumption and COVID-19 is complex. Nicotine consumption can be increased during the pandemic (13). Nevertheless, other studies found that smoking consumption might have been reduced (14). Following the first assumption, several studies have shown that high anxiety levels, stress and isolation suffered during the pandemic might have increased cigarette consumption (15). Additionally, a recent study showed that although 46.7% of smokers thought about quitting because of COVID-19, most did not change their smoking habits during the pandemic (16). On the other hand, smoking consumption might have been decreased because of perceived pulmonary potential risks, lower access opportunities and lower social interactions because of the lockdowns (17). Being scared of getting infected by COVID-19 and believing coronavirus is more severe for smokers might have decreased smoking consumption (18).

Previous systematic reviews and meta-analyses have focused on evaluating the impact of smoking on COVID-19 progression and severity. The main findings pointed out that users with any smoking history (currently or in the past) had an increased and significant risk of developing severe symptoms and worse hospital outcomes in terms of mortality, disease progression and need for medical ventilation (19–22). As far as we are, concerned there is no systematic review assessing how the COVID-19 pandemic has affected smoking consumption, which is crucial to identifying risk factors for wellbeing.

The potential effect of the COVID-19 pandemic on smoking behavior is still uncertain, and a better understanding of the magnitude of nicotine consumption because of the COVID-19 pandemic is required to predict long-term consequences on global population health. Additionally, it is crucial to help health services and policy-makers cope with the impact of COVID-19 and provide an appropriate response. Therefore, the objective of this systematic review is to analyse how the patterns of nicotine consumption changed during the COVID-19 pandemic through longitudinal studies.

## Methods

### Study Design

This systematic review was developed following PRISMA guidelines (23, 24). The protocol of this study was previously registered at PROSPERO on October 1, 2021 (PROSPERO ID: CRD42021282235). According to the protocol, a systematic review and meta-analysis study were planned. However, due to considerable (I^2^ = 92.7%; 95% CI, 916% to 95%) and significant heterogeneity between the included studies (Q_18_ = 264.13; *p* < 0.001), the meta-analysis was not performed. These analyses were performed based on Cochran's Q statistic, its *p*-value and I^2^ index and its 95% CI.

### Search Strategies

The search strategies were implemented in MEDLINE (via OVID and PubMed), ERIC (via OVID), PsycARTICLES (via OVID), Scopus, Web of Science (WOS) and PsycINFO from database inception to 24 October 2021. In addition, the reference lists of included articles were reviewed manually. The search strategy included the following three sets of relevant terms: a set of words related to COVID-19, a second set made up of a combination of words related to tobacco consumption, and the third set of words related to the design of the studies. The words included within the sets were combined with the Boolean OR, while the three sets of words were combined with the Boolean AND. All words were searched by title and abstract. The search was piloted in PubMed and then adapted to run across OVID, Scopus, WOS and PsycINFO. The search strategies in all databases are shown in Supplementary Material.

### Eligibility Criteria

The rationale for our inclusion criteria was to have an extensive assessment of all prospective cohort studies that aim to explore the effect of the COVID-19 pandemic on tobacco consumption habits. Following PICOS's acronym, we included all types of populations (P). We aimed to explore studies developed before and during the COVID-19 pandemic (I/E) regarding intervention or exposure. A comparator was not applicable (C). The outcome was a change in nicotine consumption habits (O), and the study design was exclusively prospective cohort studies. No restrictions were imposed regarding publication year, language, or study setting. Table 1 shows the inclusion and exclusion criteria.

**Table 1:** Inclusion and exclusion criteria.

**Criteria**	**Inclusion criteria**	**Exclusion criteria**
Population	All population types	None
Outcome	Impact of COVID-19 impact on smoking habits	Other outcomes
Design	Prospective cohort studies	Retrospective cohort, cross-sectional, case-control, clinical trials, systematic reviews and meta-analysis, protocols, clinical case, editors' letters, qualitative studies, randomized controlled trial, brief reports.
Publication year	All year	None
Language	All languages	None
Setting	All settings	None

### Selection of Studies

Two reviewers (NA and IGG) independently screened the studies in two phases to assess eligibility: (1) abstracts and title inspection and (2) full-text inspection. Disagreements were resolved by consensus between both reviewers (NA and IGG), and in case of disagreement, a third reviewer made the final decision. The web-based software Rayyan system was used for recording decisions (25). The initial degree of agreement between the reviewers at the abstract and title inspection phases (Cohen κ = 0.43; 95% CI, 0.227 to 0.636) and the full-text inspection phase (Cohen κ = 0.94; 95% CI, 0.810 to 1.060) was moderate and very good, respectively.

### Data Extraction

Two reviewers (NA and IGG) independently extracted all qualitative and quantitative relevant characteristics in an Excel sheet specifically created for the study. Discrepancies between reviewers were resolved by consensus. For the qualitative synthesis, first authors & publication year, region & country, target population, sex, sample size at baseline, sample size at follow-up, the main outcome, smoking consumption measure, number of smokers at baseline, number of smokers at follow-up results were extracted. The corresponding authors of the included studies were contacted to request more information when necessary.

Previous evidence suggests that the strictness of government policies taken to deal with the COVID-19 pandemic have an influence on tobacco use behaviors (26, 27). Thus, a part from the variables reported above, the COVID-19 stringency index (as a measure of country-level response to COVID-19) created by the Oxford COVID-19 Government Response Tracker (OxCGRT), was used (28) to calculate the mean daily COVID-19 stringency index. The COVID-19 stringency index uses nine criteria (school and work closures, restrictions on public gatherings, annulment of public events, closures of public transport, stay-at-home requirements, public information campaigns, restrictions on internal movements, and international travel controls) to calculate an index that ranges from 0 to 100. The higher the score, the greater the restrictive response. The mean daily COVID-19 stringency index was calculated for each study taking into account the follow-up period of each study. To do so, the daily COVID-19 stringency indexes during the periods, in which the follow-up of each study was carried out, have been sum and divided by the total number of days included in the follow-up.

### Assessment of Risk of Bias

The risk of bias in the included studies was measured independently by two reviewers (NA and IGG) using the Newcastle–Ottawa Scale for observational studies (NOS) (29). The NOS scale consists of eight items divided across three domains: (1) selection of the cohort, (2) comparability of the cohort and (3) outcome. Each study is evaluated through the assignment of stars. Each study can only receive one star for each item in the selection and outcome categories and a maximum of two stars for comparability. The stars designate the items with a low risk of bias. Thus, according to the NOS scale, the risk of bias in each study might be interpreted as follows: very high risk of bias (0–3 stars), high risk of bias (4–6 stars), and low risk of bias (7–9 stars) (30).

## Results

### Selection of the Studies

**Figure 1:**
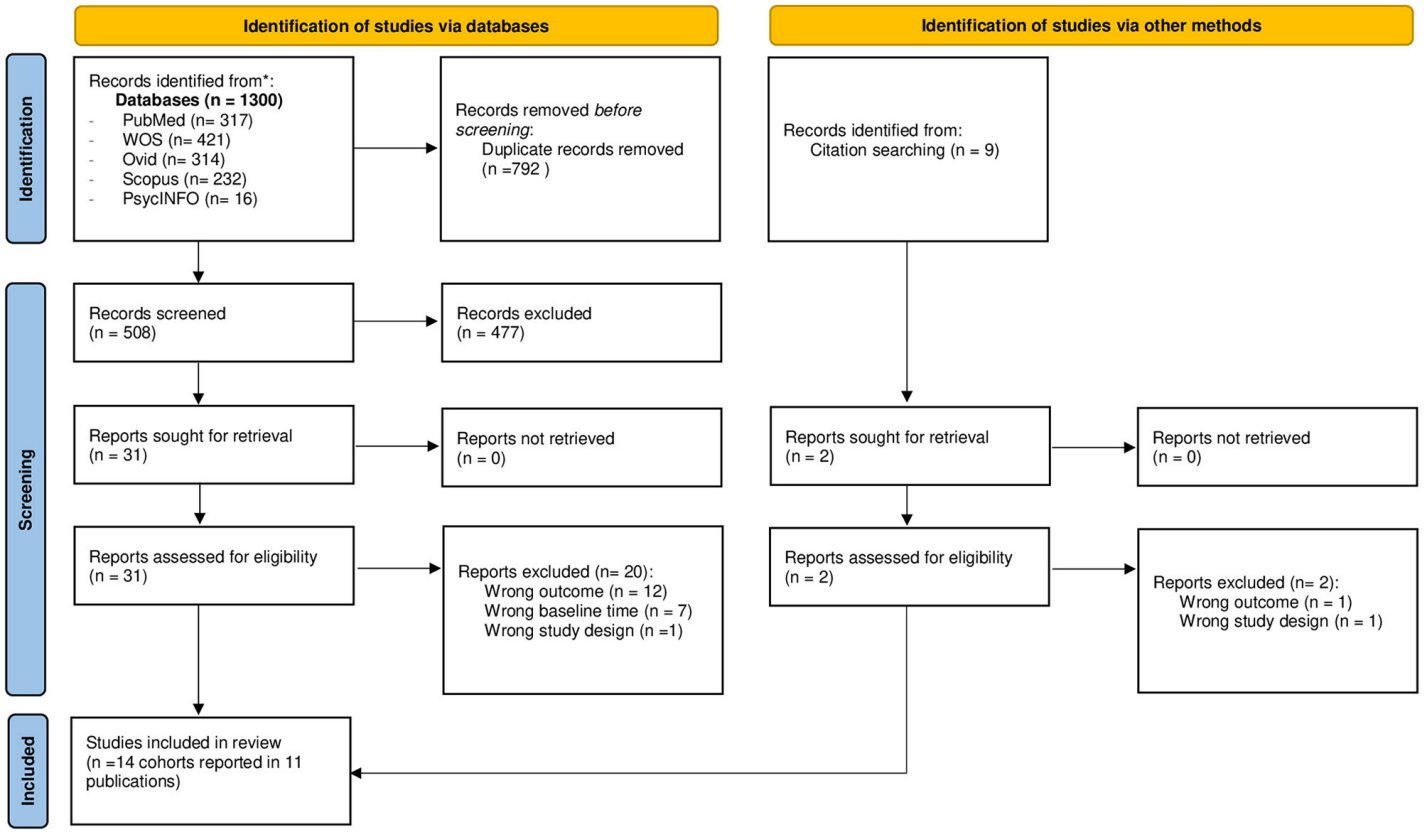
PRISMA 2020 flow diagram of the studies included.

A total of 1,300 records were found through databases, and 9 records were found through other sources (reference lists inspection of the included articles in full-text screening). After eliminating duplicates, 509 records remained and were reviewed for the title and abstract inspection. Of these, 31 studies met the inclusion criteria and were reviewed for full-text inspection. Finally, 14 cohorts reported in 11 publications met the inclusion criteria for the systematic review (see Figure 1). We must note that the study of García-Esquinas et al. (31) included four different cohort studies (ENRICA cohort study, ES cohort study, TSHA cohort study and Exernet cohort study) in the same publication.

### Characteristics of the Included Studies

Table 2 shows the qualitative characteristics of the included studies. A total of 58052 participants were included across 14 cohorts reported in 11 publications. The sample size ranged from 295 (35) to 22,823 (34) (median = 1,642). All of the included studies were published in 2021. A total of 45.5% (*n* = 5) of the studies were conducted in Europe (31, 33, 34, 37, 40), while 36.4% (*n* = 4) were conducted in the USA (32, 35, 38, 42) and 18.2% (*n* = 2) in Asia (39, 41).

**Table 2:** Characteristics of the studies included in the systematic review.

**First Authors (year)**	**Region (Country)**	**Target population**	**Sex (n; %)**	**Sample size at T0 and T1 (n)**	**Main outcome**	**Smoking consumption measure**	**N° of smokers at T0 (n; %)**	**N° of smokers at T1 (n; %)**	**Length of follow-up**	**COVID19 stringency index**
Chaffee et al. (32)	Northern California (USA)	Adolescents	F: (311; 59.7) M: (206; 39.5)	T0: 521 T1: 465	Changes in nicotine use	Self-reported nicotine consumption; Self-reported number of days using cigarettes over the past 30 days.	C: 20 (3.8) E-C: 58 (11.3)	C:19 (4.1) E-C: 50 (10.8)	T0: September–December 2019 T1: March - September 2020	65.88
Ekström et al. (33)	North-western and central parts of Stockholm (Sweden)	Young adults aged 23–26 years old	F: (996; 60.6) M: (648; 39.4)	T0: 1641 T1: 1641	Changes in smoking consumption	Self-reported smoking consumption	C: 513 (31.2)	C: 465 (28.3)	T0: 2016–2019 T1: August–November 2020	56.53
Garcia-Esquinas et al. (31) ENRICA	Madrid (Spain)	Older adults aged ≥ 65 years old	F: (655; 49.5) M: (668; 50.5)	T0: 1323 T0: 1323	Changes in smoking status	Self-reported smoking status	C: 118 (8.9)	C: 93 (7.0)	T0: 2019; T1:27 April 2020-22 June	71.44
Garcia-Esquinas et al. (31) ES	Barcelona and Madrid (Spain)	Older adults aged ≥ 60 years old	F: (265; 57.1) M: (199; 42.9)	T0: 464 T1: 464	Changes in smoking status	Self-reported smoking status	C: 76 (16.4)	C: 72 (15.5)	T0: 2019-2020 T1: T1:27 April 2020-22 June	71.44
Garcia-Esquinas et al. (31) TSHA	Toledo (Spain)	Older adults aged ≥ 65 years old	F: (499; 60.2) M: (330; 39.8)	T0: 829 T1: 829	Changes in smoking status	Self-reported smoking status	C: 56 (6.8)	C: 31 (3.7)	T0: 2019-2020 T1: 27 April 2020-22 June	71.44
Garcia-Esquinas et al. (31) Exernet	Aragón, Castilla-La Mancha and Madrid (Spain)	Older adults aged ≥ 65 years old	F: (335; 78.8) M: (90; 21.2)	T0: 425 T1: 425	Changes in smoking status	Self-reported smoking status	C: 7 (1.7)	C: 3 (0.7)	T0: 2019-2020 T1:27 April 2020-22 June	71.44
Niedzwiedz et al. (34)	All regions (UK)	Adults aged > 18 years old	F: (12607; 52.2) M: (10216; 48.7)	T0: 22823 T1: 10987	Changes in the number of cigarettes per day and weekly e-cigarette use	Self-reported smoking consumption; Self-reported number of days using cigarettes over the past 30 days; Self-reported weekly e-cigarette use	C: 3,446 (15.1) E-C: 1,118 (4.9)	C: 1,339 (12.2) E-C: 516 (4.7)	T0: 2017-2019 T1: April 2020	79.63
Papp et al. (35)	Midwestern U.S.A (USA)	Young adults aged 18-21 years old	F: (209; 70.8) M: (86; 29.2)	T0: 295 T1: 295	Changes in nicotine consumption	Self-reported nicotine use; Self-reported number of cigarettes smoked in the entire life	C: 160 (54.2)	C: 117 (39.7)	T0: September 2017 - September 2019 T1: March 27 - April 6, 2020	72.69
Pelham et al. (36)	22 study sites across the U.S.A (USA)	Adolescents from 10.5 to 14.6 years old	F: (818; 56.7) M: (3999; 51.0)	T0: 1,079 T1: 1,079	Changes in nicotine consumption	Self-reported number of days using cigarettes over the past 30 days; Self-reported number of days using electronic nicotine delivery system	C: 0 (0)	C: 16 (1.5)	T0: September 2019-January 2020 T1: August 2020	67.13
Roges et al. (37)	Central Catalonia (Spain)	High-schooled adolescents aged 14–18 years old	F: (556;51.4) M: (494; 45.7)	T0: 1,442 T1: 303	Changes in tobacco consumption	Self-reported daily smoking of tobacco	C: 156 (10.8)	C: 27 (8.9)	T0: October 2019 - February 2020 T1: June - July 2020	56.87
Romm et al. (38)	Atlanta, Boston, Minneapolis, Oklahoma, San Diego, Seattle (USA)	Young adults aged 18–34 years old	F: (818; 56.7) M: (624; 43.3)	T0: 1,082 T1: 1,082	Changes in cigarette and e-cigarette use over the past 30 days	Self-reported past 30-day use of cigarettes and e-cigarettes	C: 265 (26.1) E-C: 328 (32.3)	C: 233 (21.5) E-C: 287 (26.5)	T0: Fall 2019 T1: Spring 2020	63.62
Siddiqi et al. (39)	10 most populous cities (Pakistan)	Smokers aged > 15 years old	N/A	T0: 2,062 T1: 2,062	Changes in smoking behavior	Self-reported changes in smoking behavior	C: 2062 (100)	C: 1772 (85.9)	T0: September 2019 -February 2020 T1: May–July 2020	72.19
Thorisdottir et al. (40)	All regions (Iceland)	Post-secondary school aged 13–18-year-olds	13 years old: F: (1935; 49.6); M: (1958; 50.2); 14 years old: F: (1909; 49.6); M: 1930; 50.2); 15 years old: (F: (1849; 50.5), M: (1812, 49.4); 16 years old: F: (1757; 50.3); M: (1706; 48.8); 17 years old: F: (1571; 50.7), M: (1509; 48.7); 18 years old: F: (1464; 51.9), M: (1332; 47.3)	T0: 13 years old: (3900); 14 years old: (3846); 15 years old: (3665); 16 years old: (3494); 17 years old: (3098); 18 years old: (2819). T1: 13 years old: (3292); 14 years old: (3421); 15 years old: (3123); 16 years old: (3013); 17 years old: (2546); 18 years old: (2080)	Changes in frequency of cigarette and e-cigarette smoking over the past 30 days	Self-reported frequency of cigarette and e-cigarette smoking; Self-reported e-cigarette use in the past 30 days	C: 13 years old: [311 (8.0)]; 14 years old: [112 (2.9)]; 15 years old: [240 (6.5)]; 16 years old: [411 (11.8)]; 17 years old: [468 (15.1)]; 18 years old: [511 (18.1)]; E-C: 13 years old: [311 (8)]; 14 years old: [1,540 (14)]; 15 years old: [856 (23.4)]; 16 years old: [411 (11.8)]; 17 years old: [468 (15.1)]; 18 years old: [1372 (48.7)]	C: 13 years old: [42 (1.3)]; 14 years old: [106 (3.1)]; 15 years old: [107 (3.4)]; 16 years old: [107 (3.6)]; 17 years old: [151 (5.9)]; 18 years old: [143 (6.9)]; E-C: 13 years old: [146 (4.4)]; 14 years old: [333 (9.7)]; 15 years old: [369 (11.8)]; 16 years old: [424 (14.1)]; 17 years old: [578 (22.7)]; 18 years old: [509 (24.5)]	13–15-year-olds cohorts T0: Feb 6–8, 2018 T1: Sept 14–Nov 20, 2020 16–18-year-old cohorts T0: Oct 15–31, 2018 T1: Oct 6–Nov 20, 2020	47.79
Wang et al. (41)	Rushan, Qufu and Laoling (China)	Older adults aged ≥ 60 years old	F: (2063; 63.6) M: (1180; 36.4)	T0: 3243 T1: 2785	Changes in smoking status Changes in prevalence of smoking	Self-reported smoking status	C: 678 (20.91)	C: 549 (19.73)	T0: May 2019 T1: August - September 2020	78.24

*Sex: F, Female; M, Male; T0, Baseline (pre-COVID-19 pandemic); T1, Follow-up (during COVID-19 pandemic); N° of smokers: C, Cigarette; E-C, E-Cigarette*.

Related to the target population, four (36.4%) of the studies included adolescents (32, 36, 37, 40), three (27.3%) included young adults (33, 35, 38), two (18.2%) included adults (34, 39) and two (18.2%) included older adults (31, 41).

Five studies (45.5%) reported changes in nicotine, smoking or tobacco consumption as the main outcome (32, 33, 35–37). Three studies (27.3%) reported changes in cigarette use as the main outcome (34, 38, 40), and two studies (18.2%) reported changes in smoking status (31, 41). In addition, four studies (36.4%) reported information about e-cigarette consumption (32, 34, 38, 40).

Regarding the smoking consumption measure, all studies used self-reported questions about nicotine, smoking or tobacco consumption; smoking status; the number of cigarettes/e-cigarettes smoked over a specific period; and the number of days using tobacco products over a specific period. Of those, three studies (27.3%) reported both self-reported nicotine/smoking consumption and self-reported number of days using cigarettes or number of cigarettes smoked over a specific period (32, 34, 35).

The percentage of cigarette smokers at baseline (pre-COVID-19 pandemic) and follow-up (during COVID-19 pandemic) varied across studies. At baseline, it ranges from 0% (36) to 100% (39), while at follow-up, it ranges from 0.7% (García-Esquinas et al., (31) - Exernet cohort) to 85.9% (39). Regarding the number of e-cigarette smokers, a lower prevalence was observed than in traditional cigarettes. It ranged from 11.3% (32) to 48.7% (40) at baseline and from 10.8 (32) to 26.5 (38) at follow-up. The majority of the studies showed a reduction in cigarette and e-cigarette smokers from baseline to follow-up. Only two studies (18.2%) reported an increase in cigarette (36) or e-cigarette (40) consumption from baseline to follow-up.

Finally, the mean daily COVID-19 stringency index varied from 47.79 to 79.63 (Mean: 67.59; SD: 8.8). The two studies with the highest COVID-19 stringency index at follow-up were conducted in China (41) and the United Kingdom (34) and both of them showed a reduction in the percentage of cigarette and/or e-cigarette smokers.

### Risk of Bias of the Included Studies

The risk of bias in the included studies is shown in Table 3. All the studies had a low risk of bias in the categories of representative of the non-exposed cohort, selection of the non-exposed cohort, a demonstration that the outcome of interest was not present at the start of the study, and comparability of cohorts and adequate length of follow-up. In addition, all studies except one (37) had a low risk of bias in the adequacy of follow-up of the cohort category. Because self-reported measures were used in all of the studies, a high risk of bias was observed for the categories of ascertainment of exposure and assessment of outcome. By considering this, it can be concluded that all studies except one (37) presented a low risk of bias.

**Table 3:** Risk of bias of the included studies.

**First author (year)**	**Selection**				**Comparability**	**Outcome**				**RoB (total score)**	**RoB (categories)**
	**Representativenes of the exposed cohort**	**Selection of the non exposed cohort**	**Ascertainment of exposure**	**Demonstration that outcome of interest was not present at start of study**	**Comparability of cohorts on the basis of the design or analysis**	**Assessment of outcome**	**Adequacy of the length of follow-up**	**Adequacy of follow-up of cohorts**			
Chaffee et al. (32)	⋆	⋆		⋆	⋆	⋆		⋆	⋆	7	Low risk of bias
Ekström et al. (33)	⋆	⋆		⋆	⋆	⋆		⋆	⋆	7	Low risk of bias
Garcia-Esquinas et al. (31) ENRICA cohort	⋆	⋆		⋆	⋆	⋆		⋆	⋆	7	Low risk of bias
Garcia-Esquinas et al. (31) ES cohort	⋆	⋆		⋆	⋆	⋆		⋆	⋆	7	Low risk of bias
Garcia-Esquinas et al. (31) TSHA cohort	⋆	⋆		⋆	⋆	⋆		⋆	⋆	7	Low risk of bias
Garcia-Esquinas et al. (31) Exernet cohort	⋆	⋆		⋆	⋆	⋆		⋆	⋆	7	Low risk of bias
Niedzwiedz et al. (34)	⋆	⋆		⋆	⋆	⋆		⋆	⋆	7	Low risk of bias
Papp et al. (35)	⋆	⋆		⋆	⋆	⋆		⋆	⋆	7	Low risk of bias
Pelham et al. (36)	⋆	⋆		⋆	⋆	⋆		⋆	⋆	7	Low risk of bias
Roges et al. (37)	⋆	⋆		⋆	⋆	⋆		⋆		6	High risk of bias
Romm et al. (38)	⋆	⋆		⋆	⋆	⋆		⋆	⋆	7	Low risk of bias
Siddiqi et al. (39)	⋆	⋆		⋆	⋆	⋆		⋆	⋆	7	Low risk of bias
Thorisdottir et al. (40)	⋆	⋆		⋆	⋆	⋆		⋆	⋆	7	Low risk of bias
Wang et al. (41)	⋆	⋆		⋆	⋆	⋆		⋆	⋆	7	Low risk of bias

*RoB, Risk of bias. The stars designate the items with a low risk of bias*.

## Discussion

In this systematic review, we aimed to analyse how the patterns of nicotine consumption changed during the COVID-19 pandemic through longitudinal studies.

To the best of our knowledge, this is the first systematic review assessing the impact of COVID-19 on smoking consumption behavior. Global results of this systematic review evidenced that the pattern of tobacco use have decreased during the pandemic. Most smokers decreased the number of cigarettes and e-cigarettes consumed from baseline (before the COVID-19 pandemic) to follow-up (during the COVID-19 pandemic). Consistent with previous research, reductions in smoking consumption might be explained by less difficulty in quitting smoking and higher motivation to quit because of fear of the pandemic and COVID-19 negative progression (14, 18, 43). In this sense, the pandemic and fear of being infected by COVID-19 have motivated patients to quit or reduce smoking (44). Additionally, lockdowns worldwide and a reduction in social activities might have reduced the opportunities for social smoking or even the accessibility to buy cigarettes (45). In this line, we have to note that the studies with the highest COVID-19 mean daily stringency index presented reductions in the percentage of cigarette and/or e-cigarette smokers. According to this, United Kingdom, China (Rushan, Qufu and Laoling regions), Midwestern U.S.A (USA), Pakistan and Spain (Barcelona, Madrid, Toledo, Aragón and Castilla-La Mancha) showed the highest COVID-19 mean daily stringency index and an evidenced reduction in smoking consumption. However, a previous meta-analysis did not find an association between prevalence of smoking and mean daily stringency indexes (46).

Therefore, the evolution of the pandemic might have positively impacted population wellbeing in terms of smoking consumption.

Iceland and USA (22 study sites across the U.S.A) showed an increment in tobacco consumption in comparison with pre-pandemic level (36, 40). It is worthy to highlight the case of USA, which can show patterns variations in smoking consumption across different states. This increase might be caused by feelings of boredom during the lockdown (47). Additionally, smoking can be considered an unhealthy strategy to cope with stress, where people feel that they do not control the number of cigarettes they consume (48).

This study presents some limitations. First, following the PROSPERO register, a systematic review and meta-analysis study was planned. However, it was impossible to perform the study's meta-analytic part due to the high heterogeneity between studies. Regarding smoking status measures, all of the studies evaluated smoking status by self-reported questions. This might be explained by many of the studies assessing smoking as a secondary outcome. In addition, most of the studies collected data through online surveys due to the exceptional circumstances derived from the COVID-19 pandemic. Regarding population, we must note that many of the studies were focused on young populations. Therefore, our conclusions apply mainly to this population profile. Despite these limitations, this systematic review addressed a novel topic. To the best of our knowledge, we must note that all previous systematic reviews aimed to explore the effect of smoking on COVID-19 severity (19, 20, 49–51). Apart from that, we must note that this study explore the most relevant databases in the area combined with extensive supplementary hand searching. Moreover, many participants from different countries and continents were included, supporting its external validity. In addition, Supplementary Material about the COVID-19 pandemic situation in each country during the follow-up periods was included by using the COVID-19 stringency index. Regarding methodology, study selection, data extraction and risk of bias assessment were performed by two independent reviewers following the PRISMA statement.

## Conclusions

The impact of the COVID-19 pandemic on smoking behavior is complex and unclear. The results from this systematic review indicate that, in most cases, smoking consumption has decreased during the COVID-19 pandemic, while this global health crisis has been considered a good opportunity to reduce or quit smoking. Fear of becoming infected by COVID-19 and developing a maladaptive progression of the infection have motivated people to drop out of this habit. Lockdown and social restrictions have also played a crucial role in decreasing nicotine consumption. Nevertheless, in some cases, the pandemic has negatively affected smoking behavior. In these cases, the increase in nicotine consumption during the pandemic may be caused by boredom, stress and anxiety. These findings support the development of prevention and intervention strategies during the recovery phase of the pandemic to help people reduce smoking and avoid relapses in people who have quit.

## Data Availability Statement

The original contributions presented in the study are included in the article/Supplementary Material, further inquiries can be directed to the corresponding author/s.

## Author Contributions

NA and IG-G designed and conducted the study, prepared the initial protocol draft, and revised the manuscript. IG-G supervised the methodology. All authors read, provided feedback, discussed and approved the final manuscript.

## Funding

This study was supported by the Universidad Loyola Andalucía.

## Conflict of Interest

The authors declare that the research was conducted in the absence of any commercial or financial relationships that could be construed as a potential conflict of interest.

## Publisher's Note

All claims expressed in this article are solely those of the authors and do not necessarily represent those of their affiliated organizations, or those of the publisher, the editors and the reviewers. Any product that may be evaluated in this article, or claim that may be made by its manufacturer, is not guaranteed or endorsed by the publisher.
