# Supplementary material for: The Impact of the COVID-19 Pandemic on Smoking Consumption: A Systematic Review of Longitudinal Studies
Source: Front Psychiatry. 2022 Jul 12;13:941575. doi: 10.3389/fpsyt.2022.941575 (PMC9320170; doi:10.3389/fpsyt.2022.941575)
Supplement: Supplementary file 1 [file Data_Sheet_1.DOCX]

**Supplementary Material**

**Search strategies**

1. **Pubmed Search strategy (24.09.2021). Results: 317 documents**

((((((((("2019-nCoV"[Title/Abstract]) OR ("novel coronav*"[Title/Abstract])) OR ("Coronav* disease 2019"[Title/Abstract])) OR ("Coronav* disease COVID-19"[Title/Abstract])) OR ("COVID-19"[Title/Abstract])) OR ("COVID 19"[Title/Abstract])) OR ("COVID19"[Title/Abstract])) OR (coronavirus[Title/Abstract])) AND ((((((((smok*[Title/Abstract]) OR (tobacco[Title/Abstract])) OR (cigar*[Title/Abstract])) OR (nicotine[Title/Abstract]))) OR ("Tobacco Use Disorder"[MeSH Terms])) OR ("tobacco use"[Title/Abstract])) OR ("tobacco product*"[Title/Abstract]))) AND (((cohort[Title/Abstract]) OR (prospective[Title/Abstract])) OR (longitudinal[Title/Abstract]))

1. **Scopus Search strategy (24.09.2021) Results: 232 documents**

( ( TITLE-ABS-KEY ( smoke* ) )  OR  ( TITLE-ABS-KEY ( tobacco ) )  OR  ( TITLE-ABS-KEY ( cigar* ) )  OR  ( TITLE-ABS-KEY ( nicotine ) )  OR  ( TITLE-ABS-KEY ( "Tobacco Use Disorder" ) )  OR  ( TITLE-ABS-KEY ( "tobacco use" ) )  OR  ( TITLE-ABS-KEY ( "tobacco product*" ) ) )  AND  ( ( TITLE-ABS-KEY ( "2019-nCoV" ) )  OR  ( TITLE-ABS-KEY ( "2019 novel coronav*" ) )  OR  ( TITLE-ABS-KEY ( "Coronav* disease 2019*" ) )  OR  ( TITLE-ABS-KEY ( "Coronav* disease COVID-19" ) )  OR  ( TITLE-ABS-KEY ( "COVID-19" ) )  OR  ( TITLE-ABS-KEY ( "COVID19" ) )  OR  ( TITLE-ABS-KEY ( "COVID 19" ) ) )  AND  ( ( TITLE-ABS-KEY ( cohort ) )  OR  ( TITLE-ABS-KEY ( prospective ) )  OR  ( TITLE-ABS-KEY ( longitudinal ) ) )

1. **OVID (MEDLINE, ERIC & PsycARTICLES) Search strategy (24.09.2021) Results: 314 documents**

|  | **Search term** | **Results** |
| --- | --- | --- |
| **1** |  "smok*".ab,ti. | 325227 |
| **2** |  tobacco.ab,ti. | 112304 |
| **3** |  "cigar*".ab,ti. | 82131 |
| **4** |  nicotine.ab,ti. | 44376 |
| **5** |  "Tobacco Use Disorder".ab,ti. | 292 |
| **6** |  "tobacco use".ab,ti. | 22878 |
| **7** |  "tobacco product* ".ab,ti. | 6343 |
| **8** |  1 or 2 or 3 or 4 or 5 or 6 or 7 | 409099 |
| **9** |  "2019-nCoV".ab,ti. | 1379 |
| **10** |  "2019 novel coronav* ".ab,ti. | 1356 |
| **11** |  "Coronav* disease 2019 ".ab,ti. | 33884 |
| **12** |  "Coronav* disease COVID-19 ".ab,ti. | 4624 |
| **13** |  "COVID-19".ab,ti. | 161314 |
| **14** |  "COVID19".ab,ti. | 1500 |
| **15** |  "COVID 19".ab,ti. | 161314 |
| **16** |  9 or 10 or 11 or 12 or 13 or 14 or 15 | 166529 |
| **17** |  cohort.ab,ti. | 709891 |
| **18** |  prospective.ab,ti. | 702884 |
| **19** |  longitudinal.ab,ti. | 322244 |
| **20** |  17 or 18 or 19 | 1518530 |
| **21** |  8 and 16 and 20  ERIC <1965 to May 2021>(0) PsycARTICLES + Lippincott, Williams & Wilkins (LWW) - Revistas suscritas por CBUA(17) Ovid MEDLINE(R) ALL <1946 to September 23, 2021>(297) | 314 |

1. **Web of Science (24.9.2021) Results: 421 documents**

**Query #1**

((((((((TS=("2019-nCoV")) OR TS=("novel coronav*")) OR TS=("Coronav* disease 2019")) OR TS=("Coronav* disease COVID-19")) OR TS=("COVID-19")) OR TS=("COVID 19")) OR TS=("COVID19")) OR TS=(coronavirus)

**Query #2**

((((((TS=(smok*)) OR TS=(tobacco)) OR TS=(cigar*)) OR TS=(nicotine)) OR TS=("Tobacco Use Disorder")) OR TS=("tobacco use")) OR TS=("tobacco product*")

**Query #3**

((TS=(cohort)) OR TS=(prospective)) OR TS=(longitudinal)

**Query #4**

#1 AND #2 AND #3

1. **PsycInfo – 24.9.2021 Total de resultados = 16**

**S1**

( TI "2019-nCoV" OR AB "2019-nCoV" ) OR ( TI "novel coronav*" OR AB "novel coronav*" ) OR ( TI "Coronav* disease 2019" OR AB "Coronav* disease 2019" ) OR ( TI "Coronav* disease COVID-19" OR AB "Coronav* disease COVID-19" ) OR ( TI "COVID-19" OR AB "COVID-19" ) OR ( TI "COVID 19" OR AB "COVID 19" ) OR ( TI "COVID19" OR AB "COVID19" ) OR ( TI "coronavirus" OR AB "coronavirus" )

**S2**

( TI "smok*" OR AB "smok*" ) OR ( TI "tobacco" OR AB "tobacco" ) OR ( TI "cigar*" OR AB "cigar*" ) OR ( TI "nicotine" OR AB "nicotine" ) OR ( TI "Tobacco Use Disorder" OR AB "Tobacco Use Disorder" ) OR ( TI "tobacco use" OR AB "tobacco use" ) OR ( TI "tobacco product*" OR AB "tobacco product*" )

**S3**

( TI "cohort" OR AB "cohort" ) OR ( TI "prospective" OR AB "prospective" ) OR ( TI "longitudinal" OR AB "longitudinal" )

**S4**

(S1 AND S2 AND S3)
